# A Multi-Level, Mobile-Enabled Intervention to Promote Physical Activity in Older Adults in the Primary Care Setting (iCanFit 2.0): Protocol for a Cluster Randomized Controlled Trial

**DOI:** 10.2196/resprot.8220

**Published:** 2017-09-12

**Authors:** Y Alicia Hong, Samuel N Forjuoh, Marcia G Ory, Michael D Reis, Huiyan Sang

**Affiliations:** ^1^ School of Public Health Texas A&M University College Station, TX United States; ^2^ Department of Family Medicine Baylor Scott and White Health Temple, TX United States; ^3^ Department of Statistics Texas A&M University College Station, TX United States

**Keywords:** iCanFit, older adults, mHealth intervention, intervention development, intervention trial, primary care

## Abstract

**Background:**

Most older adults do not adhere to the US Centers for Disease Control physical activity guidelines; their physical inactivity contributes to overweight and multiple chronic conditions. An urgent need exists for effective physical activity-promotion programs for the large number of older adults in the United States.

**Objective:**

This study presents the development of the intervention and trial protocol of iCanFit 2.0, a multi-level, mobile-enabled, physical activity-promotion program developed for overweight older adults in primary care settings.

**Methods:**

The iCanFit 2.0 program was developed based on our prior mHealth intervention programs, qualitative interviews with older patients in a primary care clinic, and iterative discussions with key stakeholders. We will test the efficacy of iCanFit 2.0 through a cluster randomized controlled trial in six pairs of primary care clinics.

**Results:**

The proposed protocol received a high score in a National Institutes of Health review, but was not funded due to limited funding sources. We are seeking other funding sources to conduct the project.

**Conclusions:**

The iCanFit 2.0 program is one of the first multi-level, mobile-enabled, physical activity-promotion programs for older adults in a primary care setting. The development process has actively involved older patients and other key stakeholders. The patients, primary care providers, health coaches, and family and friends were engaged in the program using a low-cost, off-the-shelf mobile tool. Such low-cost, multi-level programs can potentially address the high prevalence of physical inactivity in older adults.

## Introduction

The benefits of regular physical activity on the well-being of older adults are well established. Even small increases in physical activity at a population level could have far-reaching positive impacts on chronic diseases such as diabetes, cardiovascular diseases, and several cancers [1-3]. The US Centers for Disease Control and Prevention and the American College of Sports Medicine have recommended 150 minutes of moderate-to-vigorous physical activity (MVPA) per week [4]; however, less than 5% of American older adults adhere to the guideline [5-7]. Physical inactivity is associated with high prevalence of overweight (60%) and chronic conditions (80%) in this population [8]. As the elderly population continues to grow—13% of the US population will be older than 65 years of age by 2020 [8]—an urgent need exists for physical activity-promotion programs that can reach a large population of older adults efficiently.

Increasing older adults’ physical activity, especially MVPA, is challenging. Literature suggests that effective physical activity-promotion programs are those built upon social and behavioral theories and practices, extend beyond the individual-level factors, and incorporate social and health care support [9-11]. When promoting physical activity in older adults, multi-level intervention programs that promote physical activity through goal setting and tracking at the individual level, social support at the interpersonal level, continuous monitoring at the health care level, and positive social norms at the community level are more likely to have sustainable effects [10-13].

More than 67% of older Americans (65 years or older) use the Internet and 42% own a mobile phone [14]; the digital divide in older adults has narrowed in the past decade [15]. The high rates of owning mobile tools suggest feasibility of mobile-based physical activity interventions. Mobile-based programs can reach a large number of patients efficiently; such programs can be easily tailored to individual needs and integrated into the health care system where electronic health records (EHRs) have been widely adopted [16-18]. In fact, a growing number of mobile-based, physical activity-promotion programs have been developed and tested in older adults [19,20]. However, recent reviews of existing programs have shown inconclusive evidence. While some studies report significant efficacy, others suggest minimal benefits, especially compared to usual care [20,21]. These programs, which typically used simple texting or short message service (SMS) text messaging to deliver reminders, had only short-term effects [22,23]. Some programs have required participants to use newly developed websites or mobile apps, thus limiting the population reach [10,21]. Despite inconclusive evidence from mobile-based, physical activity-promotion programs, reviews of existing literature have revealed some common characteristics of successful physical activity programs. They typically (1) incorporate the patient as an active participant in goal setting and tracking, (2) are based on behavioral and socioecological theory, (3) emphasize problem solving and the use of social support, and (4) provide both proactive and follow-up support [3,10,24]. Literature also documents the importance of social support, especially support from friends and family in mobile-based, physical activity-promotion programs [25].

Older adults tend to have more trust in their primary care providers (PCPs), or *general practitioners* in some countries, compared to other populations [26]. American older adults see their PCPs at least every 6 months [27]. Thus, the primary care clinics provide an ideal setting for delivering physical activity-promotion programs to older adults. Many PCPs, however, do not counsel their patients for physical activity promotion; they either have to address other medical complaints raised by the patients or they see no necessity of bringing up physical activity in the consultation with patients [28]. Our recent interviews with PCPs found that most physicians assumed patients understood the importance of physical activity and the lack of regular physical activity was due to patients’ insufficient motivation [29,30]. A recent BMJ systematic review identified only 15 trials conducted in primary care organizations and the most typical intervention was a one-time simple counseling session by a PCP or nurse [13]. More research is therefore needed to explore the efficacy of theory-guided, physical activity-promotion programs in primary care settings.

**Figure 1:**
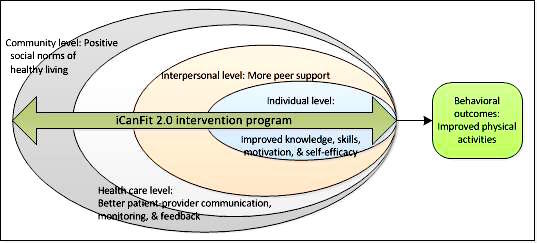
Theoretical framework of the multi-level intervention, iCanFit 2.0.

We aim to address the literature gaps noted above by proposing a multi-level, mobile-enabled, physical activity-promotion program called *iCanFit 2.0* in a primary care setting. Guided by socioecological theory, the iCanFit 2.0 program incorporates PCPs and health coaches in behavioral goal setting and continuous support for the patients. The intervention will exert effects at the individual, interpersonal, health care, and community levels. Mobile tools will facilitate patient-provider communication, enhance motivation, and provide ongoing feedback and social support to promote physical activity, as shown in Figure 1. To test the efficacy of iCanFit 2.0, we also designed a cluster randomized controlled trial (RCT) in a large health care organization.

## Methods

### Development of iCanFit 2.0

#### Overview

The development of the iCanFit 2.0 program was based on the following data sources and processes: (1) Preliminary studies with older adults in primary care settings and mHealth interventions including the iCanFit Web app, (2) qualitative interviews with older patients in a primary care clinic prior to this design, and (3) iterative discussions with stakeholders.

#### Preliminary Studies

From 2011 to 2015, we conducted the following formative research and mHealth interventions on physical activity promotion among older adults:

1. Assessment of overweight patients’ barriers to physical activity from the perspectives of PCPs. Through online surveys with 57 PCPs and focus groups with 49 PCPs, we learned that PCPs were aware of the importance of counseling older patients regarding physical activity and identified lack of motivation and social support as major barriers to regular physical activity [28-30].
2. Use of the iPod Touch for patient health behavior assessment and patient-provider communication. We developed an app on the iPod Touch—before the iPad was released—so patients could complete a brief health behavior assessment (HBA) on a touch screen while waiting for appointments with a PCP. A colorful chart report was generated instantly (see Multimedia Appendix 1). When the patient walked into the appointment with a PCP, the report became a natural conversation starter and facilitated patient-provider communication and collaborative goal setting. We piloted this app with 109 patients in a primary care clinic and the results showed that 30% of the participants reported that their PCP discussed the report with them, 24% established behavioral goals with him or her as a result of the discussion, and 90% related positive experiences with using mobile tools to generate an HBA report [31].
3. Development and testing of the iCanFit interactive website for older adults. With the goal to promote physical activity in older cancer survivors, we developed an interactive website called iCanFit. We conducted three phases of research: formative research with key stakeholders [32], usability testing of the website with target users [33], and an efficacy trial with older cancer survivors [34]. The users of iCanFit reported high levels of usefulness and satisfaction. Participants reported a higher level of quality of life (effect size=0.35) and a higher level of physical activity (effect size=0.45) following the use of the iCanFit Web app [34].

#### Qualitative Interviews With Older Diabetes Patients in a Primary Care Clinic

The initial design of iCanFit 2.0 was the combination of the HBA tablet app and the interactive iCanFit Web app. As the initial design of iCanFit 2.0 evolved, we conducted qualitative interviews with 103 older diabetes patients in a primary care clinic. Considering most older adults have multiple chronic conditions, our preliminary studies focused on different chronic conditions to ensure that iCanFit 2.0 can serve the needs of a large number of older adults. The mean age of the participants in the qualitative interviews was 50 years and the mean years of living with diabetes was 10 years. Most of these older patients used the Internet and more than half had a mobile phone. They had positive attitudes toward a mobile-based, physical activity-promotion program and offered many suggestions and concerns for the design of iCanFit 2.0.

#### Iterative Discussions With Key Stakeholders to Refine the Design

After 10 years working in healthy aging and chronic disease management, we have established rapport with local communities and health care organizations; we have also always engaged key stakeholders in the intervention design, implementation, and evaluation. In designing iCanFit 2.0, we had a series of group discussions with our key stakeholders. We brought our initial design to the meeting and obtained their feedback; the iterative process continued until a satisfactory protocol was agreed upon by all key stakeholders. The current design of iCanFit 2.0 reflects the inputs from our target patients and other key stakeholders and is substantially different from the original design.

### Community Engagement

Prior to completion of protocol design and project implementation, a Community Advisory Board (CAB) will be established, consisting of older patients, community leaders, health care providers, and administrators. At least five members of the CAB will be older patients. The CAB will have 10 members and vote on a director and a secretary. The CAB will meet with the project team every month in the first 6 months of the project and every 6 months afterward. The CAB will offer gatekeeper and stakeholder concerns as well as recommendations on program design, feasibility issues, implementation, and evaluation strategies; it will also help the research team interpret findings and advise on how to translate research findings into sustainable programs.

### Intervention Trial

#### Overview

**Figure 2:**
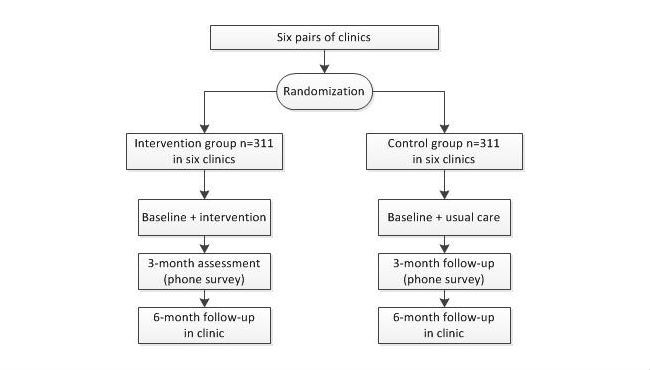
Intervention trial flowchart.

The intervention trial compares the effectiveness of the iCanFit 2.0 intervention program with a comparator program among overweight older adults in a primary care setting. To achieve this goal, we will conduct a cluster RCT in 12 family medicine clinics (six pairs) in Central Texas, USA. In each pair of comparable clinics, one will be randomized to the intervention group and one to the control group (see Figure 2).

#### Study Sites and Sources of Patients

We will conduct the proposed trial in 12 family medicine clinics that belong to a large university-affiliated, integrated health care system in Central Texas. These clinics were selected because of their similarity in patient demographics, clinic operation, and service coverage. The 12 clinics will be grouped into six pairs. Clinics in each pair have been selected to have similar characteristics of size (ie, number of patient visits per year) and number of overweight older adults as well as situated miles apart to reduce possible contamination of the intervention. We will use a randomization table to assign the two clinics in each pair into the intervention group or the control group.

#### iCanFit 2.0 Intervention Protocol

**Figure 3:**
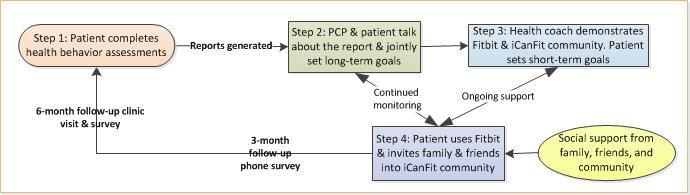
Process of iCanFit 2.0 intervention. PCP: primary care provider.

As illustrated in Figure 3, the iCanFit 2.0 intervention protocol includes four steps. First, after the eligible participants complete the informed consent forms, they will complete an HBA on an iPad (see Multimedia Appendix 1 for sample screenshots), which takes about 15 minutes. Right after completion, patients will receive a printed copy of the HBA summary report with colorful charts of their current level of physical activity compared to the recommended physical activity levels (see sample summary reports in Multimedia Appendix 1). Meanwhile, the same report will be printed out in the office of the PCP with whom the patient has an appointment. Second, the patient brings the HBA report into his/her appointment with the PCP and the report serves as a natural conversation starter to facilitate patient-provider communication and joint goal setting. Third, right after the patient’s appointment with the PCP in the primary care clinic, a health coach will meet with the patient, further explain the HBA report, and ensure the patient has set a long-term physical activity goal, if the patient has not set one with the PCP. The health coach will give the patient a Fitbit Flex 2 (Fitbit Inc) (see Multimedia Appendix 2 and section below about the device) and demonstrate how to use the device. The health coach will also help the patient to create an account on the iCanFit online community. The health coach will advise on how to set short-term (eg, weekly) goals, track and sync data, share progress with family and friends via Facebook, and obtain personalized feedback on the iCanFit online community. The counseling session will last 30 minutes; a photo-illustrative brochure with instructions on how to use the Fitbit as well as account information and reminders will be given to the patient. Fourth, the health coach will constantly monitor patients’ use of the Fitbit and iCanFit online community. Patients will receive incentives (eg, online badges, virtual coins, and honor levels) for meeting physical activity goals and updating their progress. Any questions posted on the iCanFit online community will be answered by the health coach within 12 hours. If a patient is “idle” for 2 weeks, the health coach will call the patient to offer help and address the patient’s barriers. Patients will also receive a brochure of safety tips (see Multimedia Appendix 3), which details possible adverse events during exercise and how to take action depending on the situation. For adverse events that need immediate medical attention, patients are advised to go to the nearest emergency room. For nonurgent matters, they can contact the health coach, who can assist with scheduling a clinic appointment with the patient’s PCP.

**Figure 4:**
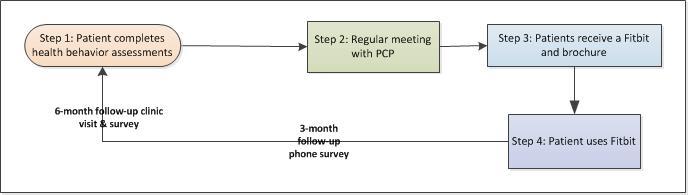
Process of comparator protocol (for patients in the control group). PCP: primary care provider.

#### Comparator

The patients in the control group will receive usual care enhanced with a Fitbit Flex 2, as shown in Multimedia Appendix 2. The Fitbit Flex 2 was released in 2016, costs US $75, and weighs a quarter of an ounce. It is compatible with iPhones and Android phones, only needs to charge once a week, syncs automatically with mobile phones within 20 feet, and easily syncs to a computer with a USB port. It tracks steps taken, stairs climbed, calories burned, distance travelled, and sleep time. Figure 4 illustrates the process of the comparator protocol. After eligible patients provide informed consent, they will first complete an HBA on an iPad while waiting for their appointment. Unlike patients in the intervention group, patients in the control group will not receive any reports from their HBA; after their appointment with the PCP, they will receive a Fitbit Flex 2 and a brochure explaining how to use the Fitbit and the benefits of regular physical activity. Patients in the control group will not be given any information about the iCanFit online community; neither will they receive counseling or monitoring from health coaches. Similar to the intervention group, control group patients will receive the safety tips of exercise brochure.

#### Recruitment of Patients

Eligibility of participants includes the following: (1) 60 years of age or older, (2) body mass index (BMI) of 25 kg/m^2^ or higher, (3) have no medical condition prohibiting regular physical activity as shown in the EHR, (4) have access to the Internet through a computer or mobile phone, and (5) have a phone that can receive SMS text messages or phone calls. Based on our prior experience of recruiting participants for mHealth interventions in primary care settings and the consideration of minimal interruption of daily operation of the clinic, we will recruit participants as follows: in the EHR system, patients who are 60 years of age or older, have a BMI of 25 kg/m^2^ or higher, and have no medical condition that prohibits them from exercise will be *flagged*. When a flagged patient checks in for his/her appointment, a health coach will be notified, who will approach the patient and explain the purpose of the project and check if the patient meets all the criteria. Eligible patients will be invited to participate in the study. Participation is completely voluntary; if the patient declines to participate, we will take notes regarding the patient’s demographic information for future analysis. If the patient agrees to participate, she/he will complete the informed consent form and be able to begin the study at the very visit when they are recruited. In case some patients agree to participate but cannot start the study at that visit, we will schedule another visit for these patients to start the study within a month.

#### Program Fidelity

We will take the following measures to ensure the program is delivered with high quality and good fidelity. First, all research staff will receive 2 months of intensive, project-specific training on research ethics, intervention design, and project implementation. They will also receive training on interviewing patients and research conduct at the family medicine clinics where the intervention will take place. Health coaches will receive additional training on how to interact with patients, how to demonstrate the use of the Fitbit Flex 2, and how to set short-term goals and track progress on the iCanFit online community. They will practice health coaching with patient representatives from our CAB until satisfactory performance is demonstrated. We will also have refresher training once a year during the project implementation. Second, health coaches will counsel patients under the supervision of a registered nurse; an experienced nurse will randomly check these counseling sessions and provide timely feedback. As part of quality control, our CAB will conduct site visits quarterly throughout the study. Third, the principal investigators of the project will work in the participating clinics for quality assurance and address any issues that may come up during the trial.

#### Patient Retention

To ensure we retain most of the patients during the intervention trial, the following measures were proposed. First, we will explain to the participants the longitudinal nature of the study during the informed consent and the fact that they will have a phone survey in 3 months and a follow-up clinic visit in 6 months. Second, 2 weeks prior to the follow-up survey or visit, we will remind the participants via SMS text message or phone call and help them schedule the appointment at their convenience. Third, for patients who do not comply with the intervention protocol, for example, do not wear the Fitbit constantly or do not sync the data in a timely fashion, we will send friendly reminders via SMS text message and motivate them with positive outcomes of physical activity and social support from the iCanFit community. Patients who continuously ignore our reminders and invitations—for 2 consecutive weeks—will be considered dropouts. We expect a 20% attrition rate at 6 months. Fourth, some participants might lose their Fitbit device during the trial. We will immediately replace a Fitbit if lost. Based on our prior experiences [35,36], less than 5% of participants may lose the mobile device during the trial. Finally, some overweight older adults may report discomfort in exercise. During the counseling by the health coach at baseline, we will advise participants to slowly increase their physical activity level and to monitor their heart rates. All participants will have a brochure of safety tips (see Multimedia Appendix 3) outlining typical adverse events and how to take appropriate actions in case of such an event. The patient is advised to go to the nearest emergency room for an event that needs immediate care. For nonurgent issues that require consultation with a PCP, the health coach will assist to schedule an appointment.

### Outcome Evaluation

#### Outcome Measures and Sources of Data

The evaluation of the iCanFit, physical activity-promotion program will be based on three datasets:

1. HBA surveys collected using the iPad at baseline and two follow-up surveys. As shown in Table 1, the survey includes demographic information, technology use and eHealth literacy [37], current level of physical activity [38], quality of life [39], patient-provider communication [40], perceived support from the health care team [41], and perceived social support for physical activity from the community [42]. All of these measures are based on validated scales with good validity and reliability. The first follow-up is a phone survey 3 months after baseline and the second follow-up is a clinic visit 6 months after baseline. This arrangement is based on the consideration that all overweight older adults are asked to visit their PCP at least every 6 months [27].
2. Fitbit data. Two types of Fitbit data will be collected for evaluation: physical activity data recorded on Fitbit and patient interactions with the health coaches and peers in the iCanFit online community.
3. Clinic data. We will collect patients’ weight and adverse events related to participating in the study from the EHRs at the clinic visits at baseline and at the 6-month follow-up.

#### Power and Sample Size Calculations

The primary outcome of the study is the total minutes of MVPA per week. MVPA is measured as “very active” and “fairly active” on Fitbit. The secondary outcome of the study is the patient’s self-report quality of life, measured by the 12-Item Short Form Survey [39]. We hypothesize the effect size of the iCanFit 2.0 to be 0.25, based on a prior Fitbit intervention trial [43]. Following the procedure developed by Cohen, the calculation of sample size is carried out in three steps by assuming a .05 significance level to achieve the power of 0.8 [44]. First, we assume that two independent samples can be obtained for the same size *n*_1_ each. Then the total sample size is *n*=2 x *n*_1_. In this ideal scenario, the total sample size *n*=398 is required to detect the effect size *d*=0.25 between the two population means. Second, because the randomization occurs at the clinic level, we need to consider the clustering effect. Previous similar research suggests a small clustering effect due to multiple patients per physician, but virtually no clustering effect between patients with different physicians in the same clinic [45]. We assume that if the intraclass correlation coefficient (ICC) per patient is equal to .05, and that ICC per physician is equal to 0, then equation 1 holds:

ICC=(1 + ICC_patient) x (1 + ICC_physican) – 1 = .05 (1)

Thus, the design effect is as follows in equation 2:

D=1 + (m - 1) x ICC (2)

where m is the average number of patients per physician in our effective sample. If we restrict m to be no more than 6, then D=1 + 5 x .05 = 1.25. We would need a sample size of 498 (ie, 398 x 1.25) after factoring in cluster effects. Third, based on our prior study of RCT primary care settings [36], we assume the attrition rate to be 0.2 after 12 months, thus the required sample size at baseline is 622 in total (ie, 498/[1-0.2]), or 52 patients per clinic on average. We will recruit no fewer than 30 and no more than 70 patients from each participating clinic.

#### Data Analysis Plan

Our analysis has been planned to correspond to the study’s main aim. We will begin with exploratory data analyses. Demographic and baseline characteristics for the participants will be summarized using descriptive statistics overall and by intervention and control groups to assess baseline comparability. Prior to analyses, we will check continuous outcome distributions and apply normalizing transformation where needed.

Our main goal is to test the hypothesis that patients in the intervention group will have more MVPA per week than the control group. To test this hypothesis, we will compare major outcomes between the intervention and control groups. We will implement multi-level regression models (eg, hierarchical linear models and mixed-effect models). Multi-level regression models are needed to account for an ICC that results from clinic-level observations [45]. Patients’ observations over time are nested within PCPs, and PCPs are nested within clinics.

Equation 3 presents an example of a multi-level regression model for a continuous outcome Y_ijt_, on clinic *i*, PCP *j*, and time point *t*. Our analyses will also include background characteristics that are not included in equation 3 for the sake of brevity. Two predictors, time (TIME) and intervention group (INTV), are classified as 1=iCanFit and 0=control. The multi-level regression model is given as follows:

Y
_ijt_ = α
_0_ + INTV
_i_ × α
_1_ + TIME
_ijt_ × α
_2_ + INTV
_i_ X TIME
_ijt_ × α
_3_ + ζ
_ij_ + ε
_ijt_ (3)

where α_0_, α_1_, α_2_, and α_3_ are the fixed effects, ζ_ij_ is a random effect (ie, random intercept) for each PCP in each clinic, and ε_ijt_ is the residual error for repeated observations over time. The random effect is assumed to be normally distributed with mean zero. The residual error is assumed to be multivariate normally distributed across repeated observations, with mean zero and a covariance matrix that models the autocorrelation among repeated observations [46]. We will examine model fit statistics to choose an appropriate covariance structure. Hypothesis testing will be carried out to test for intervention effects on the outcome of interest over time. Referring to equation 3, this is equivalent to testing the hypothesis H_0_: α_3_ = 0. If higher outcome values are desirable, then a positive significant α_3_ parameter indicates a positive intervention effect (ie, we reject H_0_: α_3_ = 0). We will use the SAS version 9.0 (SAS Institute Inc) PROC MIXED procedure and PROC GLIMMIX procedure to fit multi-level models to continuous and binary data, respectively.

We will also apply structural equation modeling (SEM) to examine the extent to which the intervention takes effect at individual, interpersonal, health care, and community levels as shown in Figure 1. Models will be constructed to measure direct and indirect effects. We will also analyze whether increased MVPA is a mediator for improved clinical outcomes. Latent variables are similar to random effects, accounting for nested observations. We will use Mplus version 3.1 (Muthén & Muthén) to fit SEM for continuous and binary data [47,48].

## Results

The proposed protocol received a high score in a National Institutes of Health review, but was not funded due to limited funding sources. We are seeking other funding sources to conduct the project.

**Table 1:** Data collected and instruments used in the survey.

Domain	Scale or indicators	Data collection mode	Number of items
Demographics	Age, gender, race/ethnicity, marital status, education, income, insurance, chronic conditions, and perceived health	Survey with patients at baseline	11
Technology use	eHealth literacy scale (E-HEALS), alpha=.78 [37]	Survey with patients at baseline	11
Physical activity	International Physical Activity Questionnaire, alpha=.76 [38]	Surveys with patients at baseline and follow-up	4
Quality of life (primary outcome)	12-Item Short Form Survey, alpha=.81 [39]	Surveys with patients at baseline and follow-up	12
Patient-physician communication	Provider Patient Communication Scale, alpha=.80 [40]	Surveys with patients at baseline and follow-up	4
Support from health care team	Patient Assessment of Chronic Illness Care (PACIC), alpha=.79 [41]	Surveys with patients at baseline and follow-up	11
Support from broader community	Perceived social support for diet and exercise, alpha=.78 [42]	Surveys with patients at baseline and follow-up	9
Total minutes of exercise per week	Total minutes of moderate-to-vigorous physical activity a week	Fitbit	Varies
Patient-provider communication	Number of questions sent by patient	iCanFit community	Varies
Patient engagement	Frequency of log-ins, syncs, and communication with iCanFit community and health coach	iCanFit community and follow-up survey	Varies
Moderate-to-vigorous physical activity (primary outcome)	Total minutes of moderate-to-vigorous physical activity a week	Fitbit	1
Overall experience with the iCanFit program	Experience of the intervention and follow-up, intention of continuing use of Fitbit and iCanFit community, and suggestions for improving the program	Follow-up survey	Varies
Secondary clinic outcomes	Weight, chronic condition, and number of sickness clinic visits and adverse events	Medical records	4

## Discussion

The iCanFit 2.0 intervention protocol has the following four strengths:

1. It is a mobile-enabled, multi-level intervention. Most existing mobile-based, physical activity-promotion programs are individual oriented and expect users to change behaviors after using the mobile tool; the complex social environment for behavioral change and maintenance has not been addressed [5,10,25]. The iCanFit 2.0 involves patients, PCPs, health coaches, and family and friends throughout the process instead of simply targeting the patients alone, thus shifting the focus from just a mobile tool to using mobile tools to foster a social environment for behavioral change and maintenance.
2. The intervention delivery is compatible with normal clinic operation. The implementation of our program is designed to fit the normal operation of the primary care clinics. For example, instead of recruiting eligible patients through mail or phone invitation [49], we will flag eligible participants in the EHR system; when these flagged patients come to visit their PCP for clinic appointments, they will be invited to participate in the study. While waiting for their appointments, patients will complete a brief HBA; immediately generated reports via the office Wi-Fi system will be sent to both patient and PCP for better patient-provider communication. Health coaches will use mobile tools for continuous monitoring, not only reducing the burden of the PCPs but also increasing service efficiency and patient outreach. Because the majority of older adults typically see their PCPs at least every 6 months [27], we set our follow-up clinic visit at 6 months. These implementation strategies will allow the proposed intervention to be sustainable and scalable.
3. The program uses the low-cost, off-the-shelf mobile device, Fitbit, for older adults. Many existing mHealth interventions have typically used newly developed mobile apps, which resulted in limited scalability and sustainability of the program [19,21]. We chose to use the Fitbit Flex 2 because of its low cost, ease of use, high compatibility, and documented reliability and validity [35,50,51].
4. The participants in the control group also receive a Fitbit Flex 2, allowing us to test the intervention effect of iCanFit 2.0 versus the mobile device alone.

The iCanFit 2.0 program also has the following limitations:

1. iCanFit 2.0 is a complex intervention with multiple components; it requires buy-in from the primary care clinics and especially the PCPs. It also requires skilled coordination and joint efforts by multiple parties.
2. iCanFit 2.0 needs well-trained health coaches for counseling and monitoring of the patients.
3. Some older patients may not have Internet access either via computers or mobile phones and, therefore, may not be able to join the study.
4. The control group does not have regular monitoring and social support and may suffer a higher rate of attrition.
5. The iCanFit online community needs active users to maintain the positive social norm, which may be challenging for older adults. It merits further study on how to further engage older adults and obtain social support online.

Despite these limitations, to the best of our knowledge, the iCanFit 2.0 intervention will be one of the first multi-level, mobile-enabled, physical activity-promotion programs for older adults in a primary care setting. It was built upon our 10 years of research of a mobile-based intervention to promote physical activity in older adults. We have engaged patients and other key stakeholders throughout the design and will continue to do so in the intervention trial.

## Appendix

Multimedia Appendix 1 Health behavior assessment screenshots and sample reports.

Multimedia Appendix 2 Fitbit Flex 2 illustration and features.

Multimedia Appendix 3 Safety tips brochure.

## Abbreviations

BMI: body mass index
CAB: Community Advisory Board
E-HEALS: eHealth literacy scale
EHR: electronic health record
HBA: health behavior assessment
ICC: intraclass correlation coefficient
MVPA: moderate-to-vigorous physical activity
PACIC: Patient Assessment of Chronic Illness Care
PCP: primary care provider
PESCA: Program to Enhance Scholarly and Creative Activities
RCT: randomized controlled trial
SEM: structural equation modeling
SMS: short message service
