# Supplementary material for: A Multi-Level, Mobile-Enabled Intervention to Promote Physical Activity in Older Adults in the Primary Care Setting (iCanFit 2.0): Protocol for a Cluster Randomized Controlled Trial
Source: JMIR Res Protoc. 2017 Sep 12;6(9):e183. doi: 10.2196/resprot.8220 (PMC5615219; doi:10.2196/resprot.8220)

Sample Screen Shots of iPad based behavioral assessment

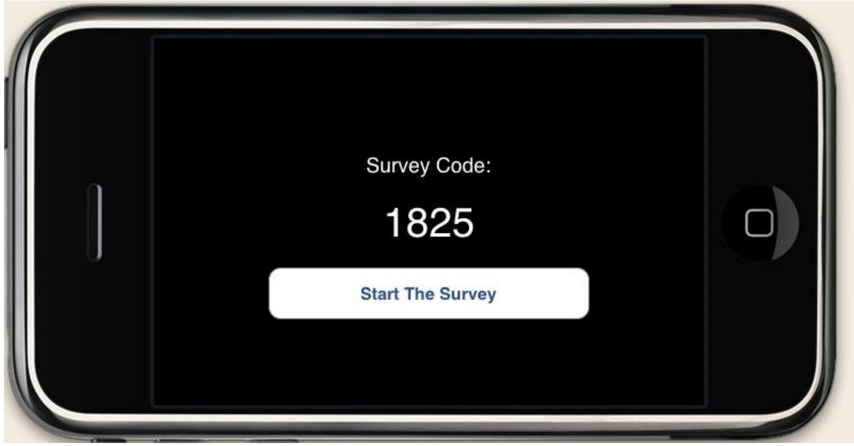

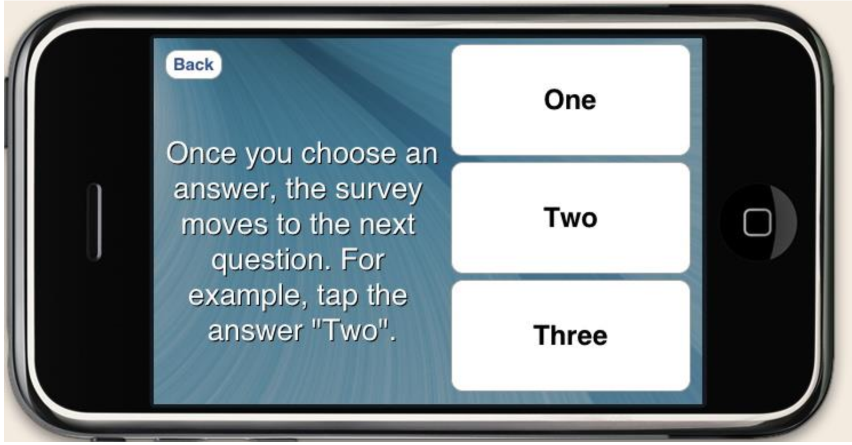

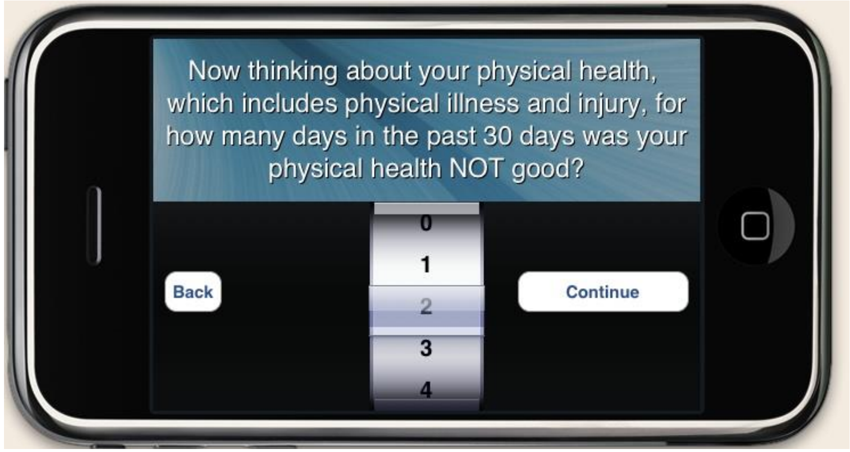

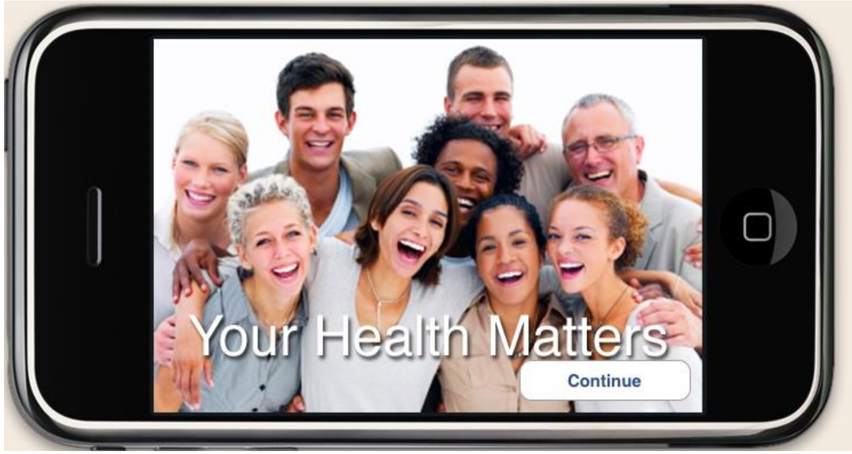

Sample Screen Shots of iPad based behavioral assessment

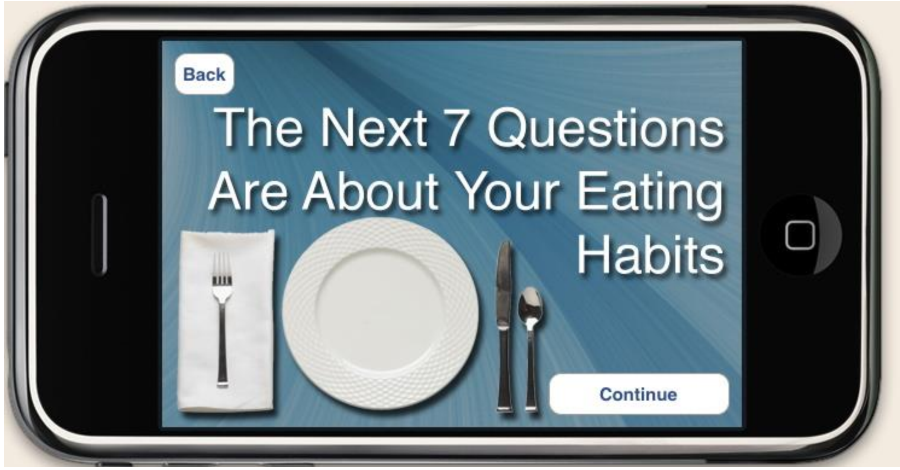

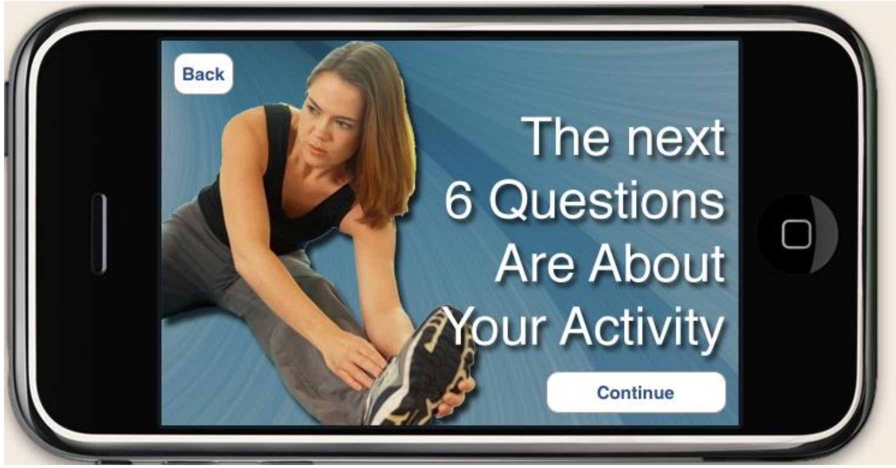

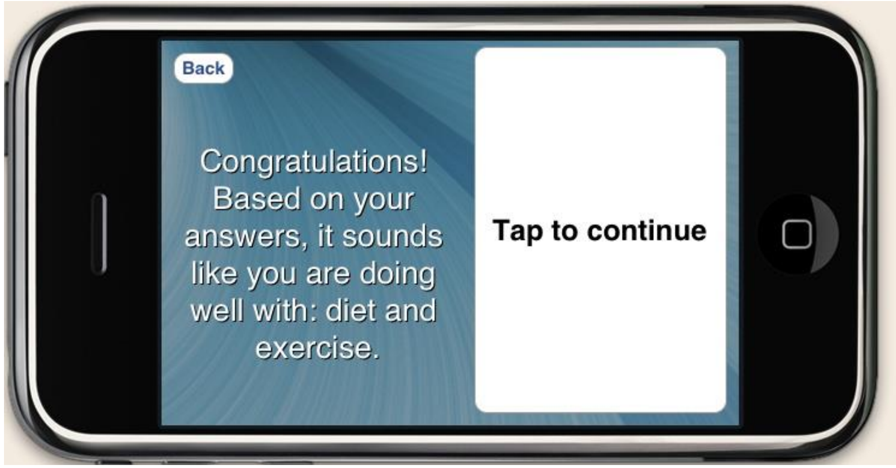

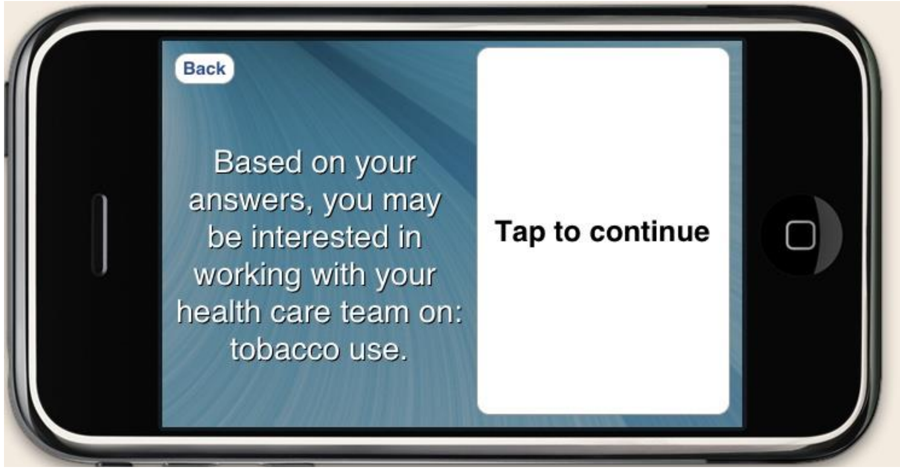

Sample: Generated health behavior assessment Report

A

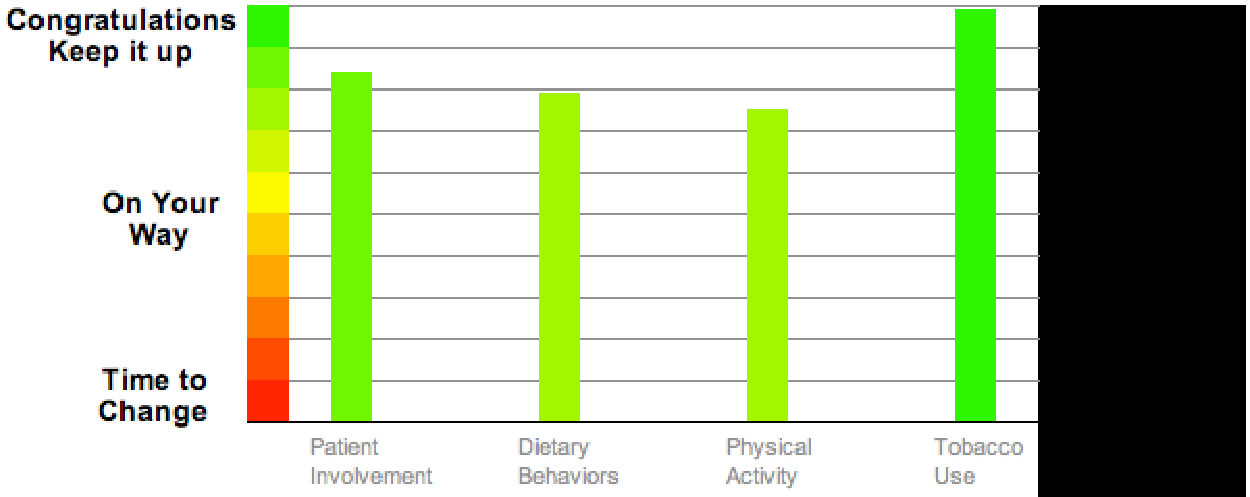

B

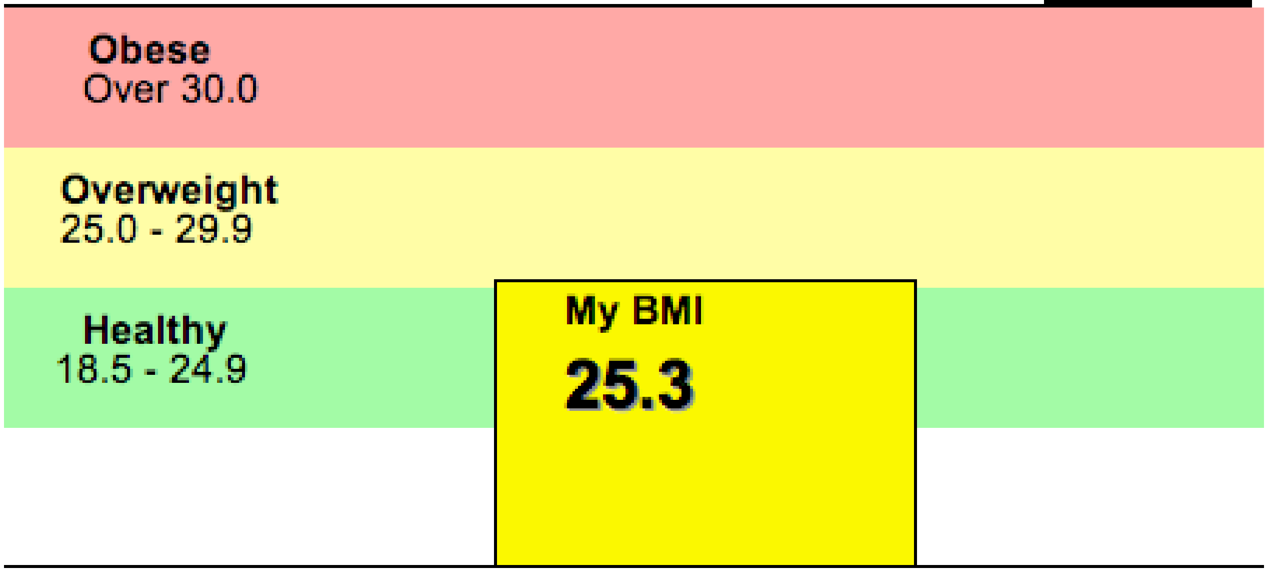

Sample: Generated health behavior assessment Report

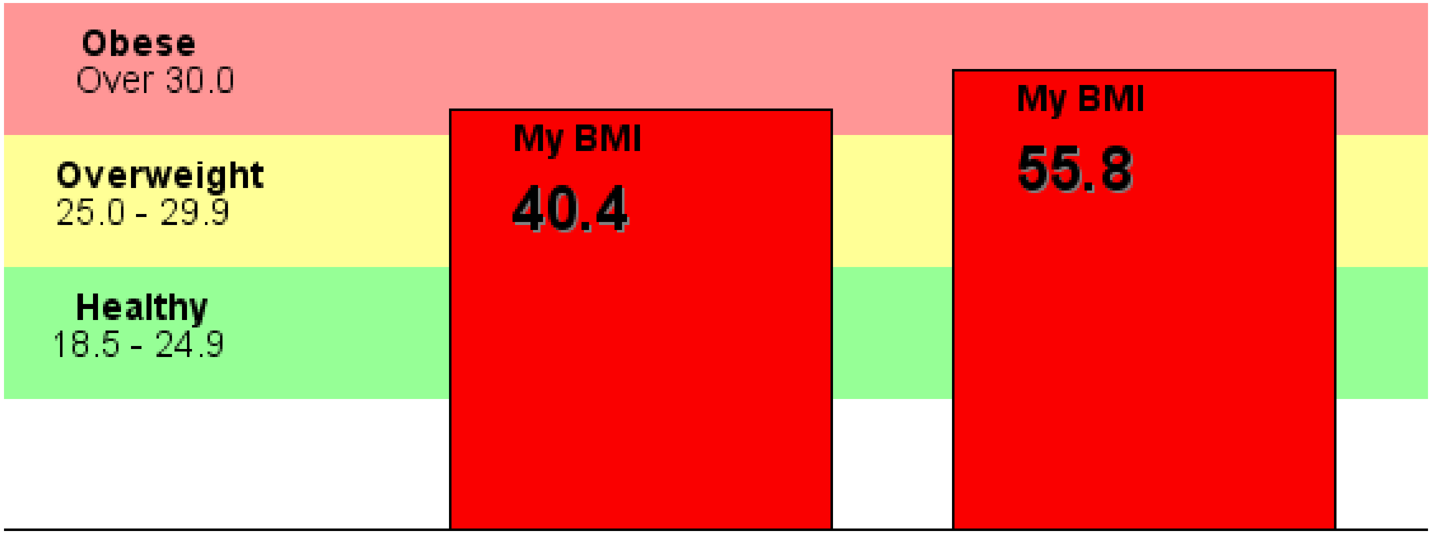

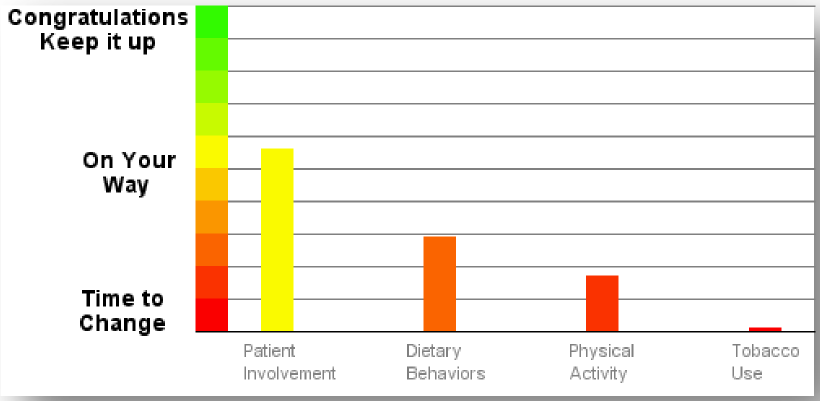

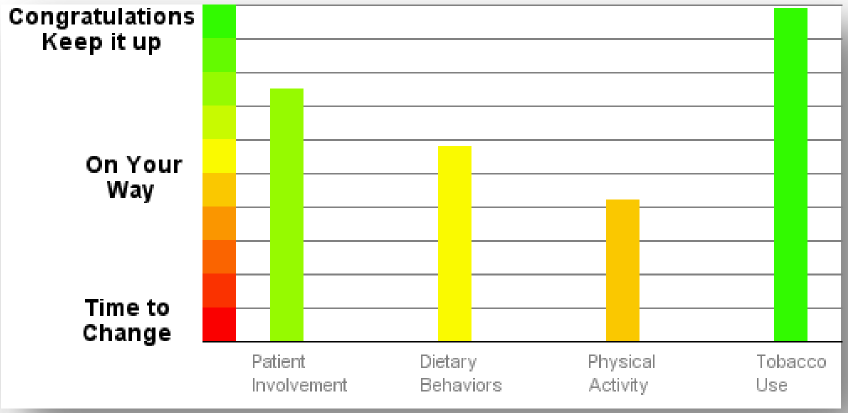

Supplement: Multimedia Appendix 1 [file resprot_v6i9e183_app1.pdf]
