# Supplementary material for: A Multi-Level, Mobile-Enabled Intervention to Promote Physical Activity in Older Adults in the Primary Care Setting (iCanFit 2.0): Protocol for a Cluster Randomized Controlled Trial
Source: JMIR Res Protoc. 2017 Sep 12;6(9):e183. doi: 10.2196/resprot.8220 (PMC5615219; doi:10.2196/resprot.8220)
Supplement: Multimedia Appendix 3 [file resprot_v6i9e183_app3.pdf]

easy
Exercise And Screening for You

Choose right- Exercise and Screening for You

www.easyforyou.info

SAFETY TIPS

Follow these EASY safety tips for when to start and stop exercise. Use the recommendations below for exercising safely with your condition.

Exercise Safety Tips to Always Consider Prior to Starting Exercise

- Always wear comfortable, loose-fitting clothing and appropriate shoes for your activity.
- Warm up: Perform a low to moderate intensity warm-up for 5-10 minutes.
- Drink water before, during and after your exercise session.
- When exercising outdoors, evaluate your surroundings for safety: traffic, pavement, weather, and-strangers.
- Wear clothes made of fabrics that absorb sweat and remove it from your skin.
- Never wear rubber or plastic suits. These could hold the sweat on your skin and make your body overheat.
- Wear sunscreen when you exercise outdoors.

Exercise Safety Tips for When to STOP Exercising

Stop exercising right away if you:

- Have pain or pressure in your chest, neck, shoulder, or arm.
- Feel dizzy or sick.
- Break out in a cold sweat.
- Have muscle cramps.
- Feel acute (not just achy) pain in your joints, feet, ankles, or legs.
- Slow down if you have trouble breathing. You should be able to talk while exercising without gasping for breath.

Exercise Safety Tips to Recognize Days/Times When Exercise Should NOT be Initiated:

- Avoid hard exercise for 2 hours after a big meal. (A leisurely walk around the block would be fine).
- Do not exercise when you have a fever and/or viral infection accompanied by muscle aches.
- Do not exercise if your systolic blood pressure is greater than 200 and your diastolic is greater than 100.
- Do not exercise if your resting heart rate is greater than 120.
- Do not exercise if you have a joint that you are using to exercise (such as a knee or an ankle) that is red and warm and painful.
- If you have osteoporosis, always avoid stretches that flex your spine or cause you to bend at the waist, and avoid making jerky, rapid movements.
- Stop exercising if you experience severe pain or swelling in a joint. Discomfort that persists should always be evaluated.
- Do not exercise if you have a new symptom that has not been evaluated by your health care provider such as pain in your chest, abdomen or a joint, swelling in an arm, leg or joint, difficulty catching your breath at rest, or a fluttering feeling in your chest.

Additional Safety Information is provided at the National Institute of Health Web page
www.nlm.nih.gov/medlineplus/safety.html

© Copyright, all rights reserved, Program on Healthy Aging, School of Rural Public Health, Texas A&M Health Science Center.
